# Further Observations on Epiphyseal Separation of the Ends of the Humerus1Read at the thirty - ninth annual meeting of the Medical Association of Central New York at Syracuse, October 15, 1906.2This communication is to supplement a paper presented by me to the Oswego County Medical Society at Pulaski, N. Y., May 9, 1899, and printed in the New York Medical Journal for September 16, 1899.

**Published:** 1907-05

**Authors:** M. M. Lucid

**Affiliations:** Cortland, N. Y.; Surgeon to Cortland Hospital


### Further Observations on Epiphyseal Separation of the Ends of the Humerus.[1 2]

By M. M. LUCID, M. D., Cortland, N. Y.
Surgeon to Cortland Hospital.

THE subject which by your courtesy I am at liberty to introduce for discussion today is one which is full of interest, and considered important alike to physician and surgeon. It has been selected by me not alone because of the paucity of research upon the conditions of which I desire to make mention, but because my attention has been attracted recently by some very unique and interesting cases and, too, because the scope of usefulness of surgical methods and appliances is continually changing and occupies the foremost place in the work, the thoughts, and the discussions of practical surgeons.

ANATOMICAL PHASES.

I shall discuss only the practical anatomical phases, the etiology, diagnosis, and treatment equitably, for the purpose of

1. Read at the thirty-ninth annual meeting of the Medical Association of Central New York at Syracuse, October 15, 1906.

2. This communication is to supplement a paper presented by me to the Oswego County Medical Society at Pulaski, N. Y., May 9, 1899, and printed in the New York Medical Journal for September 16, 1899.

reviewing such conclusions as have seemed justifiable, with the hope that I may be able to present an outline of this subject which will prove of interest, and invite your criticism.

It is, indeed, strange that so little has been written upon the subject of epiphyseal separation of the ends of the humerus, either by remote or modern surgeons; but the epiphyseal structures are small, and we are ever conscious of the fact that nowadays most surgeons are looking for larger game. The subject of epiphyseal separations is still in need of further study, and the knowledge necessary to insure positive results can only be gained by the careful study of many cases. Nothing systematic, save the brief references extended by Hamilton, Smith, and Moore, and recently by Poland, has, to the writer's knowledge, been published regarding epiphyseal separation of the ends of the humerus.

When we appreciate the positive fact that no physician or surgeon has, after improper reduction and faulty fixation of the injury referred to, had perfect movement, we have sufficient data to justify us in concluding that in no field of bone surgery is harmonious action of the physician and surgeon more necessary for the results demanded and justly due the suffering patient.

Many are the problems that present themselves for solution in the management of cases of epiphyseal separation of the ends of the humerus, for the proper determination of which all of the surgeons resources are requisite. Few cases in bone surgery make greater demands upon his tact, knowledge, experience, and judgment: or in which the necessity for intelligent and effective treatment is more urgent.

While it is not the purpose of this paper to give a detailed report of my series of cases, six of the upper and nine of the lower, in all fifteen, still I find upon consulting the literature of the subject that it is sufficiently rare to demand your attention, and from such references I feel that we can draw valuable deductions when associated with similar experiences which, no doubt, many of you have had.

A brief description of the plan of development of the humerus will enable us better to understand the separation of the epiphyses, both at the upper and lower ends of the bone. The humerus is developed or originally formed from seven cartilaginous centers—namely, one for the shaft, one for the head, one for the tuberosity, one for each epicondyle and two for the lower articulating ends of the bone. The shaft is ossified in nearly its whole length at the time of birth. Ossification begins in the centers for the upper end of the bone between the first and fourth years, and they coalesce by the end of the fifth year so as to form a single epiphysis, which finally unites with the shaft about the twentieth year of life.

At the lower end of the bone, ossification begins in the radial portion of the articular surface at the end of the second year, and the trochlear portion at the twelfth year; in the internal epicondyle at the fifth year, and in the external epicondyle at the fourteenth year. At the sixteenth or seventeenth year of life all the centers are joined to each other and to the shaft, except the inner epicondyle, which does not unite by bone with the shaft till the eighteenth year. It will be observed, therefore, that although the ossification begins in the upper epiphysis first, it is the last to form bony union with the shaft, which process is completed about the twenty-first year of life. It is hence apparent that the superior epiphysis of the humerus comprises not only the head of the bone, but likewise both tuberosities, with that portion of the bicipital groove which is not situated between the processes. Interally, it corresponds exactly to the cartilaginous surfaces of the head of the humerus, but in front, externally, and behind its line of junction with the shaft passes below the tuberosities. The supraspinatus and infraspinatus muscles are not, therefore, attached to the lower fragment, nor is there any difficulty in comprehending osseous union, for the head of the bone forms one body with the tuberosities, and is richly supplied with blood.

ETIOLOGY

Epiphyseal separation of the ends of the humerus belongs almost exclusively to the periods of youth and childhood, and is occasioned either by a direct blow or fall upon the shoulder, or by a violent wrenching of the arm, as during delivery, causing separation of the superior epiphysis; while separation of the lower epiphysis is usually the result of a fall upon the elbow, excessive adduction or abduction of the forearm with superextension, or a sudden or unexpected push of the arm which it is unprepared to resist.

PATHOLOGY

Upon dissection in these cases in the young, the head of the humerus is found broken off at the tubercles, but it remains in the glenoid cavity and does not move with the shaft. The upper end of the lower fragment projects in front, when displacement exists, and is less sharp and angular than in cases of a broken bone. Taking the head of the humerus of a subject ten years of age, parted by maceration we find the angle made by the junction of the plane projected through the anatomical neck which makes about two-fifths of the whole surface, with the plane passing below the tuberosities measures about one hundred degrees.

Separation of the upper epiphysis of the humerus will not necessarily open the shoulder joint, unless in cases of very great violence, as the capsule is firmly attached to the diaphysis. In the adult the epiphyseal line marks the upper limit of the surgical neck.

The muscles are apt to acquire their greatest tension at the moment of separation, and those whose actions are in direction with the shaft press upward, the head being set upon the shaft at an angle, and were it not for the muscles attached to the tuberosities, those which act in the direction of the shaft would have a tendency to roll the now movable head upon the glenoid surface; but, instead, the muscles attached to the tubercles contract and roll the head in the socket and produce dislocation backward of the facets, which position throws now the superior edge of the diaphysis forward and retains it there. When extension is made, the head works on the projecting angle of the diaphysis, and the tense deltoid muscle, pressing the shaft backward, will restore the rotundity of the shoulder; but the cessation of the extension finds the capsular muscles ready to roll the head out and project the diaphysis forward, and the symptoms return as soon as the extension ceases.

Of the lower epiphysis very little has yet been written concerning the pathology. The synovial membrane forms at about the fifteenth year and afterward, when it overlaps the epiphyseal line. The epiphyseal line is a little higher on the outer than on the inner side. It inclines obliquely downward and inward. The epiphysis is thinner internally than externally. The humerus is separated from its cartilaginous expansion at the condyles near the elbow joint, in which case the lower fragment would comprise the entire epiphysis, which is composed of four distinct pieces, but either or both epicondyles may remain attached to the fragment.

DIAGNOSIS

Notwithstanding the very positive statements with reference to the diagnosis of epiphyseal separation of the ends of the humerus by Sir Astley Cooper, Professor R. W. Smith, and Professor Frank H. Hamilton, one would scarcely suppose that great confusion had existed, and still exists, as regards the exact nature of fracture of the humerus at the point under discussion; but error of diagnosis does occur, and that constantly, as pointed out by Poland in his work upon "Separation of the Epiphyses." In my opinion this results from the fact that a clear conception of the change of position has not been put forth, and more than this, no method that secures reduction, save that of Moore's which is a most effectual one, has thus far been proposed. The constancy of the symptoms make this condition differ from most that occur in adjoining structures.

The chief diagnostic signs of separation of the upper epiphysis from the humerus are: first, an abrupt projection can be seen and felt on the front of the shoulder about one inch beneath the coracoid process, caused by the upper end of the lower fragment. Second, crepitus, when felt, is of a softer character than in fracture. Third, the end of the shaft is rounded and smooth, not sharp as in fracture, and has the form of a low cone. Fourth, the immediate recurrence of the deformity when the means employed for its reduction cease to be in operation. The most important practical point with reference to the diagnosis is that the accident is apt to be followed by arrest of development if not properly reduced. These diagnostic and unfailing manifestations deserve to be memorised, because they exemplify the great difficulty often experienced in diagnosticating exactly the true seat of the solution of continuity, as well as the necessity that these signs should be correctly interpreted. The *x*-ray affords, perhaps, the most positive means for diagnosis. The simple anatomical fact which affords valuable information with reference to the diagnosis of separation of the upper epiphysis is that the tuberosities of the humerus form a part of the upper fragment.

### TREATMENT

In view of such a displacement at the upper end of the humerus, the natural mode of reduction would seem to be one that would carry the shaft backward and thus restore the corresponding facets to their normal positions. The great error that is constantly made is the severe and protracted extension made to reduce the relaxation, which traction may, in a measure, cause the disappearance of the deformity; but the moment the parts are abandoned to the uncontrollable action of the muscles the deformity recurs.

The most effectual mode of reduction is by carrying, with moderate extension, the humerus forward and upward. The head will roll upon the glenoid surface in any motion of the arm until restrained by its capsule. Now then, while the humerus is still back of the central line of the body, the head is rolled upward, and long before the humerus is brought up perpendicularly, the capsule at the lower border of the head has become tense, thus holding it firm; while the humerus, being thrown up and restrained by its muscles, slides the diaphysis backward, producing a coaptation of the corresponding facets. In other words, the reduction is effected by carrying the arm forward and upward, using moderate extension to the perpendicular line with the body.

The retention is effected by moderate extension while bringing the arm down to the side, and maintaining this slight extension until dressings for the purpose of continuing it are applied. Moore's method, which consists of a Swinburn splint fastened to the outer side of the arm, resting its lower end in a strip of adhesive plaster, while the upper end projects two inches above the shoulder, with a notch through which a bandage is passed under the axilla for extension, fulfills easily and promptly the indications.

With reference to the diagnosis of separation of the epiphysis at the lower end of the humerus, it is easy,—on paper. But when the surgeon is called, enough time has usually elapsed to permit of swelling, and with an elbow-joint often as big as the calf of the leg, or bigger, and the bony landmarks covered deeply by congested and edematous flesh, I know of nothing more difficult, in reality, than to determine just the nature of the injury. The *x*-ray affords at this juncture valuable assistance.

There may be fracture alone of the humerus, ulna, or radius, or any two of these bones, or each may have therewith associated a dislocation, or a dislocation alone may be present. The dislocation is easiest of diagnosis because of abnormal rigidity, but fractures, either singly or with a dislocation, are less easily determined with exactitude. If the beginner in surgery will wrap the elbow of a skeleton in a pillow and try and examine the joint through its depths, he will approximate the difficulty of the case. Could we be sure of dislocation only, our duty is plain—namely, simply to reduce it at once, for any delay increases the difficulty and also invites a stiff joint. Again, if we felt certain of fracture alone, we should feel mentally at ease, for there need be no haste in setting a fracture if the fragments are not badly displaced, because no callus is formed for nearly a week, which time would be sufficient to allow swelling to subside, and reduction could then be exactly performed.

Unfortunately, however, the surgeon must often be in doubt, because of great swelling, as to the exact diagnosis, and a positive diagnosis obtained at the earliest possible moment has everything to do with good surgery. In this condition also the *x*-ray affords valuable and most reliable aid in early diagnosis. Under such circumstances, the practical point I am about to mention becomes of the utmost value. Put your patient under anesthesia, this being the proper procedure in all cases of fracture, unless there is some organic lesion. Now, apply an Esmarch bandage, starting at the hand and going very slowly but firmly up the forearm, over the swollen elbow joint, and continue until the armpit is reached. Leave the bandage on say for ten or twelve minutes. At the end of that time, remove it, beginning at the hand, but leaving the final few turns upon the upper end still tightly in situ. The elbow thus exposed will be pale, bloodless, and no longer swollen. All the congestion and edema are, for the time being, caused to vanish.

The diagnosis can now be made with positive ease upon the following points: the lower end of the upper fragment will be tilted forward, will be prominent, and the elbow joint pushed seemingly backward with limited flexion, but not extension of the forearm. The lower end of the upper fragment has greater width than any fracture at the base of the condyle and the line of separation is nearer the end of the bone. There is no change in the relation of the three bony points. The existence of abnormal mobility at a very low level on the humeral shaft, muffled crepitus, is very suggestive.

### REDUCTION

An easy mode of reduction is to grasp the forearm and make extension with the knee in the bend of the elbow, adjusting the fragments with the free hand. Now the remaining turns of the Esmarch bandage are removed, and, of course, the swelling promptly returns; but the surgeon has accomplished his purpose. He knows the real diagnosis, applies both an anterior and posterior angular splint, with pressure anteriorly over the seat of separation, is ready to protect his own reputation, and can forecast the outcome with some degree of accuracy.

We all know that it is early errors in exact diagnosis, with resulting unfortunate lines of treatment, which most often subject the practitioner to unfavorable results. The question of passive motion in this separation is absolutely essential to get perfect extension and flexion. This should be resorted to by the surgeon,—no one else,—about the seventh or eighth day; then about the twelfth day, and after that every other day until the twentieth day. The fragments should be held in apposition and motion given very cautiously, thus permitting a thorough understanding as to what is taking place at the seat of the separation. If after the twenty-second day complete flexion and extension cannot be produced, an anesthetic should be given and the adhesions broken up so as to allow both flexion and extension. Many times it is advisable, after provisional callus formation when the fragments are in apposition, to bandage the arm one day extended and the next day flexed. In this manner brilliant and satisfactory results may be obtained when other methods might result in ankylosis.

I am very glad to have had the opportunity to present this subject. There is no branch of surgery that is more interesting, and I know of none in the practice of which ingenuity, conservatism, and good judgment are more essential to the securing of good results.

41 MAIN STREET.

